# Biochemical Signals of Survival: A Study on Mortality Markers in Coronary Bypass Surgery Patients

**DOI:** 10.7759/cureus.65456

**Published:** 2024-07-26

**Authors:** Özlem Çakırköse, Ali Muhtaroğlu, Ersin Kuloglu

**Affiliations:** 1 Department of Cardiovascular Surgery, Giresun University Faculty of Medicine, Giresun, TUR; 2 Department of General Surgery, Giresun University Faculty of Medicine, Giresun, TUR; 3 Department of Internal Medicine, Giresun University Faculty of Medicine, Giresun, TUR

**Keywords:** postoperative outcomes, amylase, biochemical predictors, mortality markers, coronary bypass surgery

## Abstract

Background: Coronary bypass surgery remains a cornerstone treatment for advanced coronary artery disease. Identifying reliable predictors of postoperative mortality can significantly enhance patient care and outcomes. This study investigates the prognostic value of preoperative and postoperative amylase levels, creatinine, alanine aminotransferase, and aspartate aminotransferase as mortality markers in coronary bypass surgery patients.

Methods: We conducted a retrospective analysis of 343 patients who underwent coronary bypass surgery. We compared the preoperative and postoperative biochemical markers (amylase, creatinine, alanine aminotransferase, and aspartate aminotransferase) of patients who died within the first week post-surgery (n = 52) and those who survived (n = 291). Statistical analyses included chi-square tests for categorical variables, t-tests for continuous variables, and receiver operating characteristic analysis for predicting mortality.

Results: No significant difference was observed in the distribution of blood groups between deceased and surviving patients. However, significant differences were noted in gender distribution and mean ages, with higher mortality observed in older and male patients. Preoperative creatinine levels were significantly higher in patients who died compared to survivors. Postoperatively, deceased patients exhibited significantly higher levels of amylase, creatinine, alanine aminotransferase, and aspartate aminotransferase. Receiver operating characteristic analysis revealed that postoperative amylase, creatinine, alanine aminotransferase, and aspartate aminotransferase values were good predictors of mortality, with amylase being the most significant predictor.

Conclusion: This study highlights the importance of biochemical markers, particularly amylase, as predictors of mortality in patients undergoing coronary bypass surgery. The findings suggest that monitoring and managing amylase, creatinine, alanine aminotransferase, and aspartate aminotransferase levels pre- and post-surgery could improve patient outcomes. This study lays the groundwork for further research into the mechanistic links between these biochemical markers and patient survival, potentially leading to improved prognostic tools and therapeutic strategies.

## Introduction

Cardiovascular diseases stand as the principal cause of mortality worldwide, with coronary artery disease (CAD) leading the charge [1]. The advent of coronary bypass surgery has marked a significant milestone in treating CAD, offering patients a lifeline towards improved health and longevity [2]. Despite technological and procedural advancements that have refined surgical outcomes, the postoperative period remains fraught with the risk of mortality. This underscores a critical need within the medical community for robust indicators that can reliably predict outcomes and guide postoperative management [3]. Among the promising candidates for such prognostic markers are specific biochemical entities amylase, creatinine, alanine aminotransferase (ALT), and aspartate aminotransferase (AST), each reflecting different aspects of physiological and pathophysiological processes in the human body [4].

Amylase, an enzyme integral to the digestive process, is primarily synthesised in the pancreas and salivary glands. Normal serum levels range from 30 to 110 U/L. Elevated levels of amylase in the postoperative context may not only suggest pancreatic involvement but also act as a broader indicator of systemic stress or trauma [5]. ALT and AST are enzymes predominantly found in the liver, playing critical roles in amino acid metabolism. Normal serum levels for ALT are typically within 7 to 56 units/L and 10 to 40 units/L for AST. Their elevation post-surgery can be indicative of hepatic stress or damage, potentially stemming from intraoperative hypoperfusion or inflammation [6]. Creatinine, a byproduct of muscle metabolism, is primarily filtered by the kidneys, with normal blood concentrations ranging between 0.7 and 1.2 mg/dL for men and 0.5 and 1.1 mg/dL for women. Post-surgical elevations may signal renal impairment, which is a critical concern given the kidney's role in drug clearance and metabolic regulation [7].

The potential elevation of these markers following coronary bypass surgery suggests a complex interplay of factors, including direct surgical impact, the systemic inflammatory response, and specific organ stress or dysfunction [8]. This complexity provides a fertile ground for investigation, as understanding the dynamics of these markers about patient outcomes could revolutionise postoperative care. Current literature underscores the individual significance of these markers in various medical conditions, yet their collective prognostic value specific to coronary bypass surgery mortality remains underexplored [9].

Our study aims to fill this critical gap by meticulously examining the preoperative and postoperative levels of amylase, creatinine, ALT, and AST in coronary bypass surgery patients. By correlating these biochemical markers with mortality outcomes, we seek not only to enrich the clinical understanding of post-surgical recovery but also to pave the way for predictive models that could significantly enhance patient management and care. This research endeavours to contribute a novel perspective to the cardiac surgery field, advocating for a more integrated approach to patient assessment and monitoring, ultimately aspiring to elevate the standard of care and improve survival rates for CAD patients.

## Materials and methods

Patients eligible for inclusion in this study were adults (aged 18 years and older) who underwent coronary bypass surgery at Giresun Training and Research Hospital, Giresun, Turkey, between January 2021 and December 2022 and had a confirmed diagnosis of myocardial infarction due to coronary vascular occlusion. Only those with complete medical records and available preoperative and postoperative biochemical test results were included.

Our retrospective analysis included 343 patients who had myocardial infarction due to coronary vascular occlusion and underwent bypass surgery between January 2021 and December 2022. The patients were divided into two groups; Group M consisted of 52 patients who died within one week after surgery, and Group S consisted of the remaining 291 patients who survived beyond this duration.

We excluded patients who underwent emergency coronary bypass surgery without a preceding myocardial infarction and those with preoperative conditions such as end-stage renal disease or ongoing dialysis, as these could independently skew the biochemical markers being studied. In addition, individuals with known liver or pancreatic diseases were excluded to eliminate any confounding impacts on the levels of ALT, AST, and amylase. Lastly, patients who died during surgery or due to non-cardiac causes within the first week postoperatively were also excluded from the analysis.

These criteria ensured a homogeneous patient cohort and minimised confounding variables, thus enhancing the reliability and validity of the study outcomes. All patient data, including age and gender, were collected from medical records. Laboratory data included blood groups, amylase, creatinine, ALT, and AST. Biochemical parameters were analysed in venous blood drawn at 12 hours preoperatively and six hours postoperatively.

Statistical analysis

The data were analysed using the IBM SPSS Statistics for Windows, Version 26.0 (released 2019, IBM Corp., Armonk, NY). The suitability of the numerical variables of the patients for normal distribution was decided by looking at the skewness and kurtosis values. It was observed that age values were normal; amylase, creatinine, ALT, and AST values before and after surgery did not comply with standard distribution rules. The reference value in a normal distribution is ±1.96. The chi-square test was used to compare the distributions of gender and blood groups according to mortality status. Mann-Whitney U test was used to compare the preoperative and postoperative ALT, AST, CR and amylase values of the patients according to their mortality status. The Wilcoxon test was used to compare the amylase, creatinine, ALT, and AST values of the living and the ex-patients preoperatively and postoperatively. Kruskal-Wallis H tests were used to compare the amylase, creatinine, ALT, and AST values of the patients with and without ex according to their blood groups before and after the operation. Spearman correlation tests were used to analyse the relationship between the status of being ex and the amylase, creatinine, ALT, and AST values of the patients before and after the operation. The correlation coefficient was evaluated as a low-level relationship between 0.00 and 0.30, a medium-level relationship between 0.30 and 0.70, and a high-level relationship between 0.70 and 1.00. Receiver operating characteristic (ROC) analysis was performed to predict the probability of exiting with age and postoperative amylase, creatinine, ALT, and AST values. In the study, significance levels were carried out by considering 0.05 and 0.01 values.

## Results

Table 1 compares the sociodemographic characteristics of the patients according to mortality status.

**Table 1:** Comparison of the sociodemographic characteristics of the patients according to mortality status *p < 0.05, **p < 0.01, 𝜒2 = chi-square test (Categorical data), t = independent sample T-test Med: median, SD: standard deviation, Min: minimum, Max: maximum

Variables		Group M (n = 52)			Group S (n = 291)			p
		Number		%	Number		%	
Gender^𝜒^^2^	Female	19		36.5	56		19.2	0.009**
	Male	33		63.5	235		80.8	
Blood groups^ 𝜒^^2^	A	21		40.4	115		39.5	0.754
	B	7		13.5	52		17.9	
	0	18		34.6	101		34.7	
	AB	6		11.5	23		7.9	
Age^t^		Med.± SD	Mean (Min.-Max.)		Med.± SD	Mean (Min.-Max.)		
		70.56 ± 9.90	73 (45-89)		64.53 ± 9.48	64 (39-87)		0.000**

There was no significant difference between the blood group distributions of deceased and surviving throughout the study (p > 0.05). According to these results, the blood group distributions of the two groups were similar.

A significant difference was observed between the mean ages of the patients who died and those who did not (p < 0.05). The mean age of the patients who died was 70.56 years, and the mean age of the living patients was 64.53 years. According to these results, the mean age of the living patients is lower than the mean age of the patients who died.

The comparison of preoperative and postoperative amylase, creatinine, ALT and AST values of the patients according to mortality status is shown in Table 2.

**Table 2:** Comparison of the preoperative and postoperative amylase, creatinine, ALT, and AST values of the patients according to mortality status *p < 0.05, **p < 0.01, test statistic: Mann-Whitney U Test (the mean and standard deviation values, median, minimum, and maximum values of the data are given). ALT: alanine transaminase, AST: aspartate transaminase

Variables		Group M (n = 52)		Group S (n = 291)		p
		Med.± SD	Mean (Min.-Max.)	Med.± SD	Mean (Min.-Max.)	
Preoperative	Amylase	61.91 ± 24.35	56 (32-138)	59.94 ± 25.01	55 (15-159)	0.673
	Creatinine	1.1 ± 0.43	1 (0.5-2.56)	0.91 ± 0.22	0.9 (0.4-1.8)	0.001**
	ALT	35.94 ± 53.39	19 (7-309)	26.07 ± 23.69	20 (6-295)	0.649
	AST	29.73 ± 30.76	22 (5-208)	29.42 ± 22.22	22 (8-196)	0.859
Postoperative	Amylase	247.9 ± 180.74	187 (21-766)	69.53 ± 56.33	56 (3-613)	0.000**
	Creatinine	1.68 ± 0.67	1.46 (0.62-3.84)	0.93 ± 0.39	0.8 (0.1-4.2)	0.000**
	ALT	168.53 ± 208.13	69 (5-772)	28.2 ± 33.33	20 (5-312)	0.000**
	AST	229.08 ± 221.69	137.5 (5-751)	69.53 ± 56.33	56 (3-613)	0.000**

There was no significant difference between the patients' preoperative amylase, creatinine, ALT, and AST values according to mortality status (p > 0.05). However, a significant difference was observed between the preoperative creatinine values of the patients according to mortality status (p < 0.05). The mean creatinine of the patients who died before the operation was 1.1, while the mean creatinine of those who survived was 0.91. According to these results, it was observed that the mean creatinine of the patients who died before the operation was higher than that of the living patients.

A significant difference was observed between the patients' postoperative amylase, creatinine, ALT, and AST values according to mortality status (p < 0.05). According to these results, the amylase, creatinine, ALT, and AST values of the patients who died after the operation were higher than the living patients.

The comparison of preoperative and postoperative amylase, creatinine, ALT, and AST values of the surviving patients is shown in Table 3. There was no significant difference between preoperative and postoperative amylase, creatinine, ALT and AST values of living patients (p > 0.05).

**Table 3:** Comparison of preoperative and postoperative amylase, creatinine, ALT, and AST values of the living patients *p < 0.05, **p < 0.01, test statistics: Wilcoxon test (the mean and standard deviation values ​​of the data, as well as the median, minimum, and maximum values, ​​are given). ALT: alanine transaminase, AST: aspartate transaminase

Variables	Preoperative		Postoperative		p
	Med.± SD	Mean (Min.-Max.)	Med.± SD	Mean (Min.-Max.)	
Amylase	59.94 ± 25.01	55 (13-159)	69.5 ± 56.33	56 (3-613)	0.348
Creatinine	0.91 ± 0.22	0.9 (0.4-1.8)	0.93 ± 0.39	0.8 (0.1-4.2)	0.147
ALT	26.07 ± 23.69	20 (6-295)	28.2 ± 33.33	20 (5-312)	0.761
AST	29.42 ± 22.22	22 (8-196)	31.58 ± 35.41	22 (5-472)	0.764

The comparison of preoperative and postoperative amylase, creatinine, ALT, and AST values of the patients who died is shown in Table 4. A significant difference was observed between the preoperative and postoperative amylase, creatinine, ALT, and AST values of the patients who died (p < 0.05). According to these results, the preoperative amylase, creatinine, ALT, and AST values of the patients who died increased after the operation.

**Table 4:** Comparison of preoperative and postoperative amylase, creatinine, ALT, and AST values in the patients who died *p < 0.05, **p < 0.01, test statistics: Wilcoxon test (the mean and standard deviation values ​​of the data, as well as the median, minimum, and maximum values, ​​are given). ALT: alanine transaminase, AST: aspartate transaminase

Variables	Preoperative		Postoperative		p
	Med.± SD	Mean (Min. – Max.)	Med.± SD	Mean (Min. – Max.)	
Amylase	61.91 ± 24.35	56 (32-138)	247.9 ± 180.74	187 (21-766)	0.000**
Creatinine	1.10 ±0.43	1 (0.5-2.56)	1.68 ± 0.67	1.46 (0.62-3.84)	0.000**
ALT	35.94 ±53.39	19 (7-309)	168.53 ± 208.13	69 (5-772)	0.000**
AST	29.73 ±30.76	22 (5-208)	229.08 ± 221.69	137.5 (5-751)	0.000**

The comparison of preoperative and postoperative amylase, creatinine, ALT, and AST values according to blood groups of the patients who died is shown in Table 5. There was no significant difference between the preoperative amylase, creatinine, ALT, and AST values of the patients who died according to their blood groups (p > 0.05). According to these results, the preoperative amylase, creatinine, ALT, and AST values of the patients who died were similar in terms of blood groups.

**Table 5:** Comparison of preoperative and postoperative amylase, creatinine, ALT, and AST values according to blood groups of the patients who died *p < 0.05, **p < 0.01, test statistics: Kruskal-Wallis H test (the mean and standard deviation values ​​of the data, as well as the median, minimum and maximum values, ​​are given). ALT: alanine transaminase, AST: aspartate transaminase

Variables		A Group (n = 136)		B Group (n = 59)		0 Group (n = 119)		AB Group (n = 29)		p	Difference
		Med.± SD	Mean (Min. - Max.)	Med.± SD	Mean (Min.-Max.)	Med.± SD	Mean (Min.-Max.)	Med.± SD	Mean (Min.-Max.)		
Preoperative	Amylase	59.48 ± 4.64	50 (37-138)	73.6 ± 23.26	73 (49-103)	64.44 ± 25.46	59 (32-111)	52.4 ± 21.65	38 (34-78)	0.343	-
	Creatinine	1.05 ± 0.38	1 (0.5-1.97)	1.07 ± 0.15	1.1 (0.91-1.34)	1.25 ± 0.56	1.1 (0.56-2.56)	0.87 ± 0.28	0.79 (0.61-1.41)	0.275	-
	ALT	36.38 ±50.73	22 (7-248)	31.43 ± 2.65	21 (16-79)	36.35 ± 71.22	19 (9-309)	38.5 ± 36.46	23.5 (11-107)	0.532	-
	AST	27.88 ±1.17	22 (5-109)	47.43 ± 1.02	22 (12-208)	22.74 ± 12.03	19 (9-57)	35.33 ± 22.31	28.5 (19-77)	0.421	-
	Age	71.76 ± 9.54	74 (45-84)	65.14 ± 9.99	68 (50-79)	70 ± 11.3	70.5 (46-89)	74.33 ± 3.98	75.5 (67-78)	0.371	-
Postoperative	Amylase	243.83 ± 170.75	189 (39-766)	299.6 ± 197.99	170 (152-593)	233.07 ± 220.33	129 (21-687)	252.4 ± 98.71	307 (98-327)	0.537	-
	Creatinine	1.62 ± 0.53	1.47 (0.62-2.8)	1.97 ± 0.82	1.84 (1.21-3.15)	1.72 ± 0.81	1.51 (0.76-3.84)	1.43 ± 0.48	1.41 (1-2.21)	0.764	-
	ALT	227.51 ± 46.45	128.75 (10-772)	90 ± 92.42	72.5 (5-266)	128.73 ± 190.35	35 (19-637)	169.8 ± 197.99	68 (19-498)	0.389	-
	AST	239.11 ± 22.16	195.15 (5-751)	323.57 ± 253.82	261.5 (69-628.4)	206.82 ± 237 110	(13-723)	146.4 ± 131.15	78 (19-305)	0.542	-

There was no significant difference between the postoperative amylase, creatinine, ALT, and AST values of the patients who died according to their blood groups (p > 0.05). According to these results, the postoperative amylase, creatinine, ALT, and AST values of the patients who died were similar according to the blood groups.

Table 6 compares the living patients' preoperative and postoperative amylase, creatinine, ALT, and AST values according to blood groups. There was no significant difference between the preoperative amylase, creatinine, ALT, and AST values of living patients according to blood groups (p > 0.05). According to these results, the preoperative amylase, creatinine, ALT, and AST values of the living patients were similar according to blood groups.

**Table 6:** Comparison of preoperative and postoperative amylase, creatinine, ALT and AST values of living patients according to blood groups *p < 0.05, **p < 0.01, test statistics: Kruskal-Wallis H test (the mean and standard deviation values ​​of the data, as well as the median, minimum and maximum values, ​​are given). ALT: alanine transaminase, AST: aspartate transaminase

Variables		A Group (n = 136)		B Group (n = 59)		0 Group (n = 119)			AB Group (n = 29)		p	Difference
		Med.± SD	Mean (Min. – Max.)	Med.± SD	Mean (Min. – Max.)	Med.± SD	Mean (Min. – Max.)		Med.± SD	Mean (Min. – Max.)		
Preoperative	Amylase	56.64 ± 21.43 53	(13-122)	60.42 ± 22.53	55 (26-122)	63.94 ± 28.75		59.5 (15-159)	57.91 ± 28.4	50 (22-140)	0.382	-
	Creatinine	0.91 ± 0.22	0.90 (0.4-1.6)	0.89 ± 0.21	0.9 (0.5-1.4)	0.92 ± 0.24		0.90 (0.5-1.8)	0.9 ± 0.21	0.8 (0.6-1.3)	0.982	-
	ALT	27.95 ± 31.89	20 (6-295)	26.98 ± 16.72	21 (7-80)	23.5 ± 16.11		18 (7-109)	25.96 ± 15.73	23 (7-61)	0.443	-
	AST	30.55 ± 21.26	22 (8-116)	31.63 ± 28.06	23.5 (9-196)	27.98 ± 21.48		20 (9-137)	25.09 ± 13.98	22 (10-68)	0.476	-
	Age	63.48 ± 9.65	63 (41-87)	65 ± 7.54	64 (51-84)	64.57 ± 10.36		64 (39-87)	68.57 ± 7.72	69 (54-85)	0.086	-
Postoperative	Amylase	68.31 ± 51.28	55 (3-337)	59.4 ± 27.82	55 (19-128)	78.4 ± 73.68		59 (12-613)	59.52 ± 31.99	58 (30-176)	0.524	-
	Creatinine	0.91 ± 0.36	0.8 (0.1-2.6)	0.87 ± 0.27	0.8 (0.5-1.6)	0.97 ± 0.48		0.8 (0.4-4.2)	0.92 ± 0.26	0.9 (0.6-1.5)	0.636	-
	ALT	27.31 ± 35.33	18 (5-312)	29.29 ± 29.02	23 (7-205)	27.52 ± 26.21		19 (5-153)	33.17 ± 55.39	23 (5-282)	0.440	-
	AST	30.07 ± 26.37	21 (5-167)	32.6 ± 25.64	24 (10-131)	32.1 ± 47.4		22 (10-472)	34.52 ± 34.11	24 (9-142)	0.773	-

There was no significant difference between the living patients' postoperative amylase, creatinine, ALT, and AST values according to their blood groups (p > 0.05). These results showed that the living patients' postoperative amylase, creatinine, ALT, and AST values were similar according to their blood groups.

Table 7 examines the relationships between the deceased's status and the patients' preoperative and postoperative amylase, creatinine, ALT, and AST values. There was no correlation between the status of being ex and the patients' preoperative ALT, AST and amylase values (p > 0.05). However, a low-level positive correlation was found between the status of being ex and the patients' age and preoperative creatinine values (p < 0.05). According to these results, it was observed that if the patients' age and preoperative creatinine values increased, the possibility of the patients to be ex increased.

**Table 7:** Examination of the relationship between the status of being ex and preoperative and postoperative amylase, creatinine, ALT, and AST values of the patients *p < 0.05, **p < 0.01, r: correlation coefficient, ALT: alanine transaminase, AST: aspartate transaminase

Preoperative		Ex	Postoperative		Ex
Amylase	r	0.023	Amylase	r	0.448**
	p	0.674		p	0.000
Creatinine	r	.178**	Creatinine	r	0.441**
	p	0.001		p	0.000
ALT	r	0.025	ALT	r	0.398**
	p	0.649		p	0.000
AST	r	-0.010	AST	r	0.442**
	p	0.859		p	0.000
Age	r	.238**	-	-	-
	p	0.000	-	-	-

A moderate positive correlation was found between the deceased status and the patients' postoperative amylase, creatinine, ALT and AST values (p < 0.05). According to these results, if the postoperative amylase, creatinine, ALT, and AST values of the patients increase, it is seen that the probability of exiting will increase.

**Figure 1:**
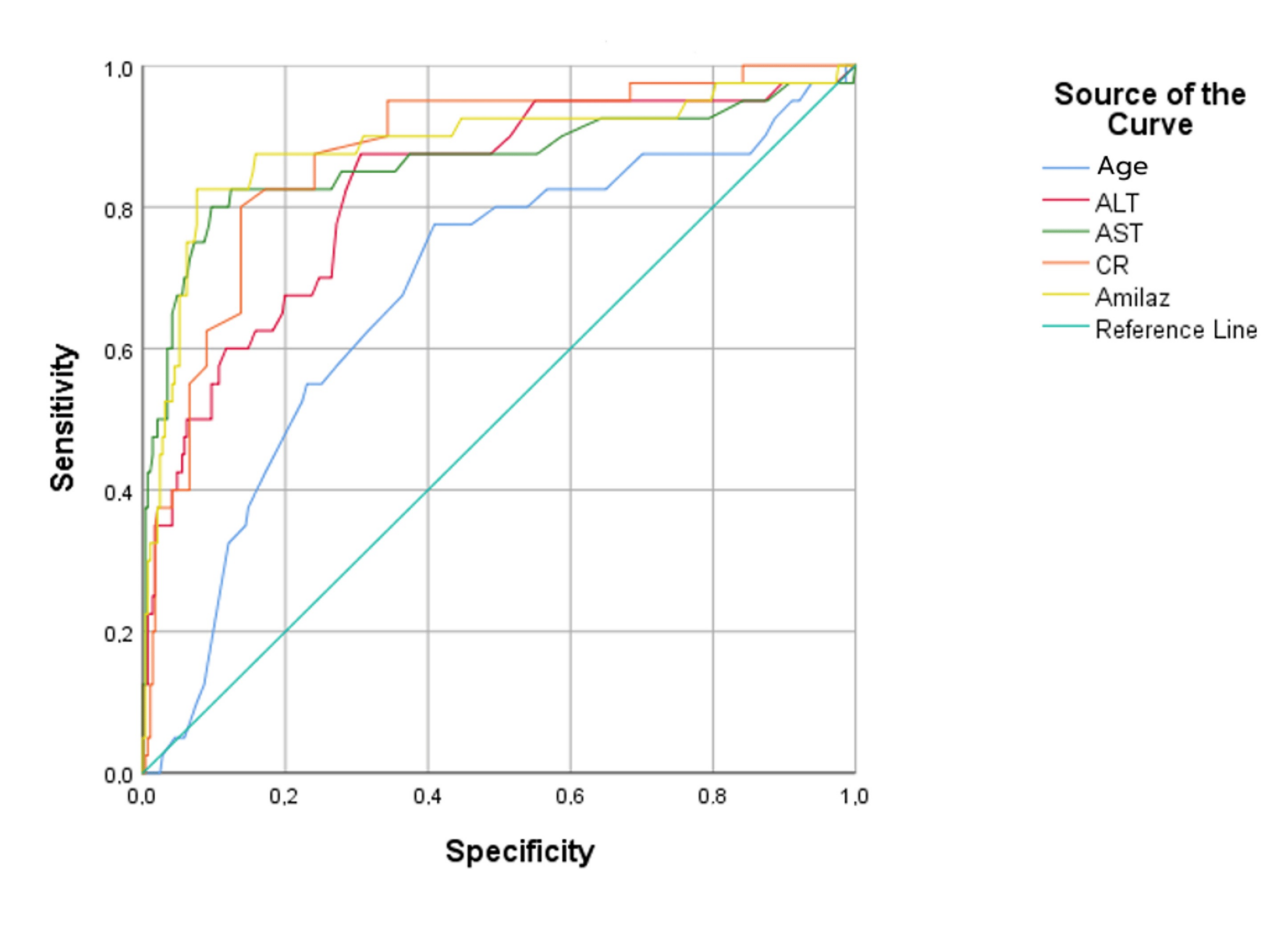
Estimation of the probability of patients being ex (diagonal segments are produced by ties). In a perfect test, these values are expected to be 1.00. In interpreting the values under the curve, 0.90-1.00 = excellent, 0.80-0.90 = good, 0.70-0.80 = moderate, 0.60-0.70 = poor and 0.50-0.60 = failure.

ROC analysis was performed to predict the probability of exiting from various parameters of the patients. The result of the ROC analysis performed to predict the probability of exiting the patients with age and postoperative amylase, creatinine, ALT and AST values is shown in Figure 1.

Table 8 shows the area under the curve, sensitivity, and specificity results obtained from the ROC analysis for predicting the likelihood of the patients' deaths with age and postoperative amylase, creatinine, ALT, and AST values.

**Table 8:** ROC analysis results for predicting the probability of patients' exiting with various parameters *AUC: area under the curve, ALT: alanine transaminase, AST: aspartate transaminase

Test variables	Area*	Std. error	p	Confidence interval (95)	
				Lowest	Highest
Amylase	0.886	0.036	0.000**	0.815	0.958
Creatinine	0.876	0.029	0.000**	0.818	0.933
ALT	0.825	0.038	0.000**	0.751	0.9
AST	0.865	0.042	0.000**	0.784	0.947
Age	0.682	0.046	0.000**	0.591	0.773

In this study, the size of the area under the ROC curve is statistically important in predicting the probability of the patients dying. The expected value of the area under the ROC curve is 0.50 when there is no ability to discriminate the probability of patients exiting as a result of ROC analyses.

In this context, age was poor in predicting the probability of exiting, while postoperative amylase, creatinine, ALT, and AST values were found to be good and significant in predicting the probability of exiting (p < 0.05). The most effective prediction parameter was found to be amylase values.

## Discussion

Our retrospective analysis of biochemical markers in coronary bypass surgery patients unveils intriguing associations between preoperative and postoperative parameters and mortality outcomes. Notably, the elevation of preoperative creatinine levels among patients who later succumbed to mortality underscores the critical role of renal function in predicting postoperative complications and survival rates [10-12]. This is consistent with the studies by Brown et al. and Thakar et al., who reported a higher incidence of adverse events such as acute kidney injury and prolonged hospital stay in patients with pre-existing renal failure undergoing cardiac surgery [13,14]. The observed disparity in postoperative outcomes based on preoperative creatinine levels emphasises the importance of preoperative renal assessment in risk stratification and personalised patient care [15].

Furthermore, the significant postoperative increase in amylase, ALT, and AST levels among deceased patients highlights the systemic stress and organ injury associated with adverse surgical outcomes [16,17]. Hepatic and pancreatic complications characterised by elevated liver enzymes and amylase levels have been associated with postoperative morbidity and mortality in cardiac surgery in studies by Yadav et al. and Kalas et al. [18,19]. These findings underscore the need for vigilant monitoring and early intervention to mitigate the impact of organ dysfunction on postoperative recovery.

The discriminatory power of postoperative biochemical markers, particularly amylase, in predicting mortality risk is a notable finding with significant clinical implications. Paik et al. and Adamu et al. demonstrated the prognostic value of amylase in various medical conditions, including acute pancreatitis and sepsis [20-22]. The robust association between elevated postoperative amylase levels and adverse outcomes underscores its potential as a valuable tool for risk stratification and early intervention in coronary bypass surgery patients.

Our study's ROC analysis further underscores the utility of biochemical markers as prognostic indicators, with amylase emerging as a standout predictor of mortality risk. These findings echo the growing interest in biomarker-based risk stratification models, advocating their integration into clinical practice to enhance patient care and outcomes. Future studies exploring the additive value of combining multiple biomarkers in predictive algorithms could unlock new avenues for personalised medicine in cardiovascular surgery.

Study limitations

Despite the strengths of our study, including a robust analytical approach and a sizeable patient cohort, several limitations warrant acknowledgement. The retrospective nature of our analysis limits causal inference and the assessment of longitudinal outcomes. Future prospective studies with extended follow-up periods and multi-centre collaborations are essential to validate our findings and elucidate the mechanistic underpinnings of biochemical markers in postoperative mortality prediction.

## Conclusions

Our study illuminates the potential of biochemical markers, particularly amylase, creatinine, ALT, and AST, as prognostic indicators in coronary bypass surgery. We have unveiled critical associations between these markers and mortality outcomes by meticulously analysing preoperative and postoperative parameters, underscoring their relevance in risk assessment and patient management. Integrating these markers into clinical practice holds promise for refining risk stratification strategies and optimising patient outcomes as we navigate the complex landscape of cardiovascular surgery. By embracing innovation and collaboration, we can usher in a new era of personalised medicine, where predictive biomarkers pave the way for tailored interventions and improved surgical care.
